# Tree Age Distributions Reveal Large-Scale Disturbance-Recovery Cycles in Three Tropical Forests

**DOI:** 10.3389/fpls.2016.01984

**Published:** 2017-01-05

**Authors:** Mart Vlam, Peter van der Sleen, Peter Groenendijk, Pieter A. Zuidema

**Affiliations:** ^1^Forest Ecology and Forest Management Group, Wageningen University and ResearchWageningen, Netherlands; ^2^Marine Science Institute, The University of Texas at Austin, Port AransasTX, USA; ^3^Instituto Boliviano de Investigación ForestalSanta Cruz de la Sierra, Bolivia; ^4^Departamento de Botánica, Escola Politécnica Superior, Universidade de Santiago de CompostelaLugo, Spain

**Keywords:** dendroecology, forest disturbance, regeneration failure, tree age distribution, tree regeneration, tree-rings, tropical forest

## Abstract

Over the past few decades there has been a growing realization that a large share of apparently ‘virgin’ or ‘old-growth’ tropical forests carries a legacy of past natural or anthropogenic disturbances that have a substantial effect on present-day forest composition, structure and dynamics. Yet, direct evidence of such disturbances is scarce and comparisons of disturbance dynamics across regions even more so. Here we present a tree-ring based reconstruction of disturbance histories from three tropical forest sites in Bolivia, Cameroon, and Thailand. We studied temporal patterns in tree regeneration of shade-intolerant tree species, because establishment of these trees is indicative for canopy disturbance. In three large areas (140–300 ha), stem disks and increment cores were collected for a total of 1154 trees (>5 cm diameter) from 12 tree species to estimate the age of every tree. Using these age estimates we produced population age distributions, which were analyzed for evidence of past disturbance. Our approach allowed us to reconstruct patterns of tree establishment over a period of around 250 years. In Bolivia, we found continuous regeneration rates of three species and a peaked age distribution of a long-lived pioneer species. In both Cameroon and Thailand we found irregular age distributions, indicating strongly reduced regeneration rates over a period of 10–60 years. Past fires, windthrow events or anthropogenic disturbances all provide plausible explanations for the reported variation in tree age across the three sites. Our results support the recent idea that the long-term dynamics of tropical forests are impacted by large-scale disturbance-recovery cycles, similar to those driving temperate forest dynamics.

## Introduction

Disturbances are increasingly being recognized as important drivers of tropical forest dynamics (e.g., Newbery et al., 2013; Tanner et al., 2014). Early studies on tropical forests focused on the importance of gap-phase dynamics, caused by frequent small-scale disturbances resulting from the death of one or a few trees, typically affecting several hundreds of square meters of forest (Denslow, 1980; Brokaw, 1985). More recent studies have highlighted the importance of infrequent disturbances that impact several hectares to square kilometers at the same time, but occur only at decadal or centennial scales (Burslem et al., 2000; Baker et al., 2005). These large-scale disturbances in tropical forest include prehistoric anthropogenic land clearings (Willis et al., 2004), multi-hectare blowdowns (Nelson et al., 1994; Vandermeer et al., 2000), fire (Baker and Bunyavejchewin, 2009) and extreme droughts (Allen et al., 2010).

Decades of research in temperate forest ecosystems have demonstrated that infrequent, large-scale disturbances are critical determinants of forest composition, structure, and dynamics (Oliver, 1980; Oliver and Larson, 1996), resulting for example in highly irregular diameter distributions of the dominant trees (Lorimer, 1980). In addition, there is a growing recognition of the heterogeneity of disturbance regimes in temperate forests and the prevalence of mixed severity disturbances and disturbance regimes (e.g., Eschtruth and Battles, 2014). Likewise, large-scale disturbances in tropical forests do not always have a high-intensity. For example, landscape-scale fires of low intensity (Baker and Bunyavejchewin, 2009) or region-wide tree mortality following extreme drought events (Nepstad et al., 2007; Allen et al., 2010) are both extensive disturbances with highly heterogeneous patterns of tree mortality and subsequent recruitment. A few studies have demonstrated the occurrence of severe and broad disturbances in tropical forests on a millennial time scale, through genetic analyses and pollen research (e.g., Daïnou et al., 2010; Lebamba et al., 2012). Forest monitoring studies on the other hand are unlikely to capture the dynamics caused by infrequent severe and/or large-scale disturbances because they are generally conducted in small permanent plots for several decades (e.g., Baker et al., 2004; Clark et al., 2010). As a result, the occurrence and consequences of disturbances that take place at an intermediate landscape scale and a temporal scale of centuries has remained understudied so far (Zuidema et al., 2013). Tree-ring research may provide information at this intermediate scale, as has been shown for temperate forests (Lorimer, 1980; Fraver et al., 2008), and to a lesser extent tropical forests (Baker et al., 2005; Middendorp et al., 2013; Nock et al., 2016).

We studied forest areas in three countries that were known to have experienced distinct anthropogenic and natural disturbances: severe droughts (Cameroon; Newbery et al., 2004), recurrent low-intensity understory fires (Thailand; Baker et al., 2008) and large-scale anthropogenic conversion in pre-Colombian times (Bolivia; Paz-Rivera and Putz, 2009). These disturbances are expected to have left marks in the current forest stand structure. In our study, we evaluate evidence for such legacies from the age distribution of shade-intolerant tree species. For each study area, we obtained age distributions of four tree species (long-lived pioneers and partial shade-tolerants) in large (140–300 ha) areas. A standardized sampling design allowed us to make comparisons among sites. To this end, we examined whether tree regeneration rates of the 12 shade-intolerant species were continuous or irregular over the past two centuries and use these regeneration patterns to reconstruct disturbance regimes.

## Materials and Methods

### Study Area and Species

In Bolivia, samples were collected in the logging concession ‘La Chonta’ (15.84° S, 62.85° W). The forest in La Chonta is a semi-deciduous moist forest (Peña-Claros et al., 2012) and Fabaceae, Arecaceae, and Moraceae are the dominant families (Toledo et al., 2011). Elevation ranges between 298 and 436 m above sea-level. Soils have been classified as relatively fertile ultisols due to human influences (Peña-Claros et al., 2012). Total annual precipitation in the region is around 1580 mm, with a 5-month dry season receiving <100 mm precipitation per month from May to September and mean annual temperature is 24.5°C. Like most of the forests in the Amazon, the area has been selectively logged at low intensity for the commercially valuable broad-leaf mahogany (*Swietenia macrophylla*). This logging took place around 1992 (Pinard et al., 1999), but we have found no direct evidence (e.g., old skid trails or tree stumps) indicating past logging in our study area. On the other hand there is abundant evidence for anthropogenic dark earths or ‘terra preta’ in the area, indicating human presence until 300–400 years BP (Paz-Rivera and Putz, 2009).

The site in Cameroon (5.25° N, 9.10° E) is a semi-deciduous tropical rainforest of the Guineo-Congolian type. Samples were collected in the logging concession 11.001 operated by Transformation REEF Cameroon (TRC). This concession is adjacent to the northwest border of Korup National Park and the forest is dominated by Caesalpinioideae, Euphorbiaceae, and Scytopetalaceae (Chuyong et al., 2004). Elevation is approximately 100 m above sea-level. No detailed information on soil characteristics is available for the site, but soils at nearby Korup are described as sandy and nutrient poor (Newbery et al., 1997). Mean total annual rainfall is around 4000 mm and mean annual temperature 26.7 °C (Nchanji and Plumptre, 2001; Groenendijk et al., 2014). The climate is characterized by a distinct 3-month dry season from December to February with monthly rainfall levels <100 mm. Of the three sites, TRC probably has the highest human influence, as several villages are located directly adjacent to the logging concession.

The third site is situated in the Huai Kha Khaeng Wildlife Sanctuary (HKK), Thailand (15.60° N, 99.20° E). The site at HKK is a semi-deciduous tropical moist forest and dominated by trees in the Annonaceae and Dipterocarpaceae family. Elevations in HKK vary between 490 and 650 m above sea-level and the soils are highly weathered slightly acidic ultisols (Bunyavejchewin et al., 2009). Mean annual rainfall is ∼1470 mm (1993–2001) and the 4–6 months dry season ranges from November to April. Mean annual temperature is 23.5°C. There is no human influence in HKK, except for the Wildlife Sanctuary infrastructure and agricultural areas around the park providing a potential ignition source for surface fires (Baker et al., 2008). No logging activities are known to have taken place in the study area.

### Field Sampling

We collected stem disks and increment cores of 1154 individual trees from the 12 shade-intolerant species listed in **Table 1**. At each site, we selected four long-lived pioneer or partial shade-tolerant species, because establishment of these species is indicative for past canopy disturbance. Besides shade-intolerance, main species selection criteria were presence of clearly identifiable annual rings and adequate local abundance enabling a sample size of approximately 100 trees. For convenience, we refer to all species by genus, except for the two *Brachystegia* species in Cameroon (*Brachystegia cynometroides* and *B. eurycoma*).

**Table 1:** Characteristics of the 12 study species, their shade-tolerance guild (LLP, long-lived pioneer; PST, partial shade-tolerant), and leaf phenology (D, deciduous; and BD brevi-deciduous).

Country	Species	Family	Ecological guild^1^	Wood density^2^ (kg/m^3^)	Leaf phenology^3^
Bolivia	*Schizolobium amazonicum*	Fabaceae	LLP	450	D
	*Sweetia fruticosa*	Fabaceae	LLP	820	BD
	*Cariniana ianeirensis*	Lecythidaceae	PST	360	D
	*Hura crepitans*	Euphorbiaceae	PST	370	D
Cameroon	*Daniellia ogea*	Fabaceae	LLP	550	D
	*Terminalia ivorensis*	Combretaceae	LLP	500	D
	*Brachystegia cynometroides*	Fabaceae	PST	620	D
	*Brachystegia eurycoma*	Fabaceae	PST	620	D
Thailand	*Afzelia xylocarpa*	Fabaceae	LLP	820	D
	*Melia azedarach*	Meliaceae	LLP	480	D
	*Toona ciliata*	Meliaceae	LLP	470	D
	*Chukrasia tabularis*	Meliaceae	PST	630	BD

*^1^Ecological Guilds: Bolivia (Peña-Claros et al., 2008), Cameroon (Hawthorne, 1995), Thailand (Herwitz, 1993; Elliott et al., 2002; Kaewkrom et al., 2005; So et al., 2010) and the definitions are in accordance with (Poorter et al., 2006); ^2^Wood density: Bolivia (van der Sande et al., 2015), Cameroon (Cirad Forestry Department, 2008; Lemmens et al., 2012), Thailand (Nock et al., 2009). ^3^Leaf phenology: Bolivia (Mostacedo et al., 2003), Cameroon (Hawthorne, 1995; Lemmens et al., 2012), Thailand (Williams et al., 2008).*

All wood samples were collected over a period of 2 years between May 2010 and May 2012. We collected samples using a clustered design in which trees were sampled within a 50 m radius of randomly assigned locations. The total area in which trees were sampled following this clustered sampling design ranged from 144 ha in Cameroon (16 clusters), to 272 ha in Bolivia (22 clusters) and 296 ha in Thailand (23 clusters). Due to the rarity of some target species, the full sample size could not be achieved with this clustered sampling scheme. For those species, we supplemented our collections with a full census of the total plot area and collected samples from all individuals (**Table 2**). All species in Bolivia, the two *Brachystegia* species in Cameroon and *Chukrasia* in Thailand were collected using the clustered sampling design; a full census was used for the other species. In Thailand *Afzelia, Melia*, and *Toona* were sampled by systematically searching the entire 296 ha area. In Cameroon *Daniellia* and *Terminalia* were sampled in two additional areas, because densities of these two species were extremely low in the previously assigned 144 ha area.

**Table 2:** Characteristics of measurements of the 12 study species.

Country	Species	*N*	*n*_rand_ (%)	*n*_missed_ (rings)	*H*_max_ (m)	*dbh*_max_ (cm)	*Age*_max_ (year)	*Age*_med_ (year)	*JG*_mean_ (mm year^-1^)	>5 cm dbh (year)	*Age- shape*
Bolivia	*Schizolobium amazonicum*	87	76	-	28.6	58.8	23	13	23.5	4	UM
	*Sweetia fruticosa*	105	35	6	27.0	71.5	220	59	3.2	26	ED
	*Cariniana ianeirensis*	102	62	7.5	37.4	125.0	170	63	4.6	17	LD
	*Hura crepitans*	97	67	3.5	34.0	216.0	163	35	8.2	11	ED
Cameroon	*Daniellia ogea*	104	*Full*^∗^	11	51.0	158.0	282	159	2.8	36	UM
	*Terminalia ivorensis*	62	*Full*	3.5	44.0	220.0	188	115.5	12.6	7	UM
	*Brachystegia cynometroides*	122	98	10	55.0	155.0	200	113.5	3.7	27	UM
	*Brachystegia eurycoma*	124	94	6	47.8	130.0	190	98	3.2	36	UM
Thailand	*Afzelia xylocarpa*	98	*Full*	6	37.9	121.2	243	67	6.6	13	UM
	*Melia azedarach*	90	*Full*	2	41.0	98.1	121	39.5	19.5	4	UM
	*Toona ciliata*	60	*Full*	4	43.0	116.4	156	57.5	6.3	10	UM
	*Chukrasia tabularis*	103	43	6	40.8	91.4	189	73	3.9	23	UM

*^∗^For these species we applied a full census of the area. Number of trees included in this study (*n*); percentage of trees sampled according to the random sampling scheme (*n*_*rand*_); median number of estimated rings missed to the pith for trees that did not include the pith (*n*_*missed*_); maximum adult stature (*H*_*max*_); maximum adult diameter (*dbh*_*max*_); maximum age (*Age*_*max*_); median age (*Age*_*med*_); mean juvenile (<20 cm dbh) dbh-growth rate (*JG*_*mean*_); years needed for 75% of the individuals to reach 5 cm dbh (>5 cm dbh); shape of the age distribution (*Age-shape*): exponential decrease (ED), logistic decrease (LD), unimodal (UM).*

The locations of all sample trees were mapped with a hand-held GPS and we measured diameter at breast height (dbh; 1.3 m) and diameter at sampling height of each sample tree. Height measurements of all sampled trees were obtained using a digital hypsometer (Nikon Forestry 550). In the logging concessions in Bolivia and Cameroon a large fraction of the wood samples for tree-ring analyses were collected as stem disks from recently felled trees. All other samples were collected using a Suunto (Vantaa, Finland) or Haglöf (Långsele, Sweden) increment borer. At a height of approximately 1 m, we manually extracted tree cores with a diameter of ∼5 mm. Therefore, ‘tree age’ and ‘year of establishment’ always refer to the time since reaching sampling height of 1 m. Depending on the diameter of the tree we used borers with lengths varying between 40 and 70 cm. From trees <40 cm dbh two cores were taken, because the borer would go straight through the tree and two radii per core were obtained; for all larger individuals we took three cores. Collecting multiple cores and stem disks allowed us to measure rings over at least three complete radii, thereby correcting for radial differences in diameter increment. We only cored trees >5 cm to minimize damage to the juveniles. Throughout this paper, the term regeneration refers therefore to the establishment of individuals into the stage of small trees, sapling or poles, of >5 cm dbh.

### Tree-Ring Measurements

Increment cores were glued to wooden mounts and cut perpendicular to the ring boundaries with a large sliding microtome (Gärtner and Nievergelt, 2010). Stem disks were sanded with progressively finer sand paper. Digital images (1600 dpi) of the tree cores and stem disks were acquired using a high-resolution flatbed scanner (Epson Expression 10000 XL) and analyzed with the program WinDENDRO (version 2009b; Regent Instruments Canada Inc.). Tree-ring boundaries were marked manually on the screen and measured to the nearest 0.016 mm (for ring structures see: Supplementary Figure S1). Large stem disks that could not be scanned, approximately 10% of the samples, were measured to the nearest 0.01 mm using a LINTAB 5 measurement device and TSAP software (Rinntech). All ring-width series were visually cross-dated within trees and then among trees. Cross-dating within trees was successful, cross-dating among trees proved, however, more difficult (Groenendijk et al., 2015). We were able to construct chronologies for the four Thai species (Vlam et al., 2014b), but not for the Bolivian and Cameroonian species (Groenendijk et al., 2014). We stress that our study was not aimed at, nor designed to, establish chronologies, we included for example many small (juvenile) trees which can typically be poorly cross-dated.

The annual character of the rings was verified by various means. For three Bolivian species, *Cariniana, Hura*, and *Sweetia*, scars in the wood produced by a fire were used to confirm the annual nature of the rings (Brienen and Zuidema, 2003; Lopez et al., 2012). The annual nature of *Schizolobium* rings was determined by counting rings of trees of known age in a plantation forest ∼200 km from the study area (P. van der Sleen, *unpublished results*). To verify dating accuracy of three Cameroonian tree species we used radiocarbon dating (Worbes and Junk, 1989). This revealed that dating accuracy of *B. eurycoma* and *Daniellia* rings was high, but dating accuracy of *B. cynometroides* rings revealed a mean error of around 10% (Groenendijk et al., 2014). This dating error in *B. cynometroides* was presumably caused by the erroneous interpretation of true rings as false rings, leading to underestimation of ages. The annual nature of *Terminalia* rings was proven by a cambial wounding experiment (Detienne et al., 1998). For all four Thai species the annual nature of the rings was also proven by a cambial wounding experiment in HKK (Baker et al., 2005). The annual growth periodicity was further supported by strong correlations between *Afzelia, Chukrasia, Melia*, and *Toona* tree-ring chronologies and seasonal climate data (Vlam et al., 2014b).

### Age Dating Accuracy

In the case that none of the stem cores included the inner most part of the stem (hereafter referred to as ‘pith’), the distance to the pith was estimated by the degree of arcing in the oldest visible ring, assuming a circular growth pattern using the protocol described by Splechtna et al. (2005). If no arcing was visible on the cores we calculated the missing distance to the pith based on the diameter at sample height measured in the field. The missing distance to the pith was then used to estimate tree age by dividing the missing radial distance to the pith by the average width of the five oldest visible rings. In 70% of the sampled trees in Bolivia the pith was visible, including all *Schizolobium* trees (**Table 2**), and hence the exact age of the tree could be retrieved. For the Cameroonian sample trees 41% could be exactly dated to the pith. For the Thai site, where sampling was restricted to coring, an exact age could be obtained for 23% of the trees based on a visible pith. Overall the number of missed rings to the pith in the cores was small, with an estimated median of six missed rings for those trees that did not include the pith (*n* = 629). **Table 2** gives an overview of the median number of rings missed to the pith for those trees in which none of the samples included the pith. For five trees the estimated missing distance was >100 rings and these trees were subsequently removed from the dataset.

Ring identification errors have likely occurred and dating errors have accumulated toward earlier dates (i.e., older trees). In particular, dating errors for *B. cynometroides* of around 10% (cf. Groenendijk et al., 2014) may have diffused the observed regeneration pattern, though were unlikely to affect the general picture. Additional dating uncertainty resulted from the missing distance to the pith in a considerable fraction (55%) of our tree-ring samples. Further aging errors may have resulted from variation in time to reach sampling height (∼1 m). Each of these dating issues has resulted in a spread in tree ages around their actual age, potentially obscuring patterns of episodic regeneration.

### Data Analysis

**FIGURE 1:**
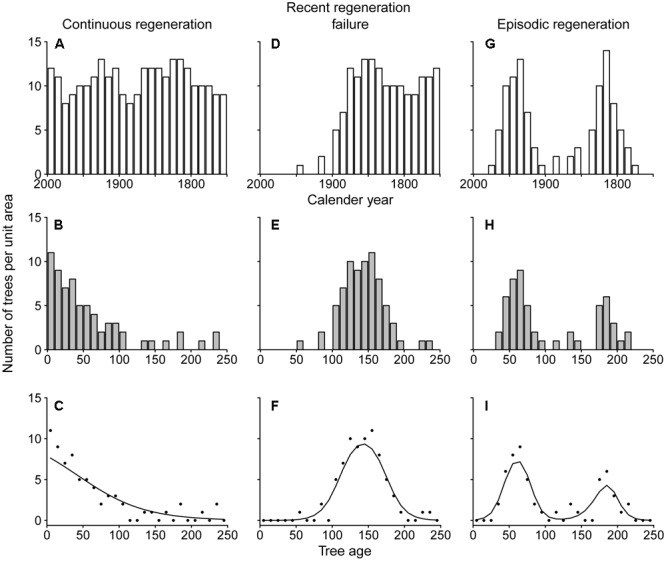
**An illustration of how temporal patterns in recruitment rate (A,D,G)** determine tree age distributions **(B,E,H)** and how these distributions can be analyzed statistically **(C,F,I)**. Temporal patterns of recruitment were generated for an imaginary shade-intolerant tree species with continuous recruitment **(A–C)**, recent regeneration failure **(D–F)** and episodic regeneration **(G–I)**. The lower abundance of old trees **(B–H)** is due to mortality, which was assumed to be 1% annually in this example. Statistical functions **(C,F,I)** are from a set of seven logistic regression functions (Jansen and Oksanen, 2013).

We used the tree-ring derived age distributions of shade-intolerant tree species to explore historical patterns of tree regeneration. To illustrate how age distributions may be interpreted, **Figure 1** shows expected age distributions of an imaginary shade-intolerant tree species for three situations and how these distributions can be analyzed statistically. In a theoretical situation of a constant disturbance rate, regeneration of shade-intolerant trees is expected to be constant over time. Under such a scenario (**Figure 1A**), age distributions are often well described by a ‘reversed-J’ shape (**Figure 1B**), assuming a constant annual mortality rate (Agren and Zackrisson, 1990; Westphal et al., 2006). In the situation of a severe disturbance followed by a period without any major disturbances, the regeneration rates of shade-intolerant species will be high for a marked time window that follows the disturbance and then drop again (**Figure 1D**, “Recent regeneration failure”). This results in a unimodal age distribution of extant trees (**Figure 1E**), where the left drop in the age distribution is hypothesized to result from lower regeneration rates and the right drop results from accumulated mortality. In the case of recurrent severe disturbances, multiple regeneration episodes may occur, and therefore multiple peaks in the age distribution (**Figure 1H**).

Age distributions of all species were analyzed to detect irregularities in regeneration rates. For all 12 species population age distributions were produced using 10-year age classes. Because sampling was limited to trees >5 cm dbh, we calculated the number of years it took trees to reach 5 cm dbh. For each species we produced an ‘age at 5 cm dbh distribution’ and we took the 75th percentile as a threshold value for all further analysis. We chose a cut-off of 75% because it results in a conservative estimate of the time needed for an individual tree to reach 5 cm dbh, while the value does not become strongly affected by a minority of persistently slow-growing individuals.

To analyze tree regeneration rates over time we used a hierarchical set of seven models (Huisman et al., 1993; Jansen and Oksanen, 2013). In this analysis, age classes were used such that in all species non-zero counts spanned at least 10 age classes (with a maximum of 29). As a result, 11 out of 12 species had age classes that were set at the standard 10 years. However, the 10-year age class bins were not suitable for some of the shorter-lived species, for which we used narrower age class bins. For *Melia*, counts per 5-year age class were used and for *Schizolobium* counts per 2-year age class were used. The seven models are logistic regression functions of increasing complexity that are traditionally used to relate species presence or abundance to environmental variables. In our case, these models were used to relate density per age class to the age data assuming a Poisson distribution and using maximum likelihood estimation. The simplest model that sufficiently explained the observed age pattern was selected based on the Akaike Information Criterion corrected for small datasets (AICc) (Jansen and Oksanen, 2013). If best-fit models were a straight line, exponential decrease or logistic decrease, we determined that regeneration rates had been relatively constant over time (**Figure 1C**; exponential decrease). If selected models were unimodal (two model types), we determined that there was evidence for high past recruitment rates and/or recent recruitment failure (**Figure 1F**). A bimodal shape (two model types) would indicate a significantly bimodal age distribution, indicative for episodic regeneration (**Figure 1I**). All statistical analyses were conducted and graphically depicted using the R Software environment version 3.0.0 (R Core Team, 2013) and the package *eHOF* (Jansen and Oksanen, 2013).

## Results

### Tree Characteristics

Maximum height, dbh and age of the sample trees were highest for Cameroon, with a median tree age of over 100 years (**Table 2**; Supplementary Figures S2E–H). Sample trees at the site in Bolivia had a comparatively lower height, dbh (Supplementary Figures S2A–D,I–L) and age, whereas the site in Thailand was intermediate between Cameroon and Bolivia.

**FIGURE 2:**
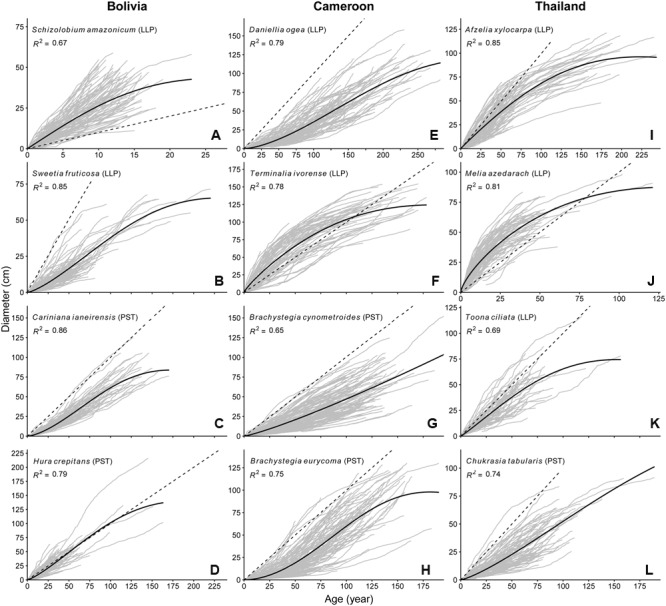
**Age diameter relation for the 12 study species.** Gray lines represent the lifetime growth trajectory for each individual tree. Dashed black line indicates a 1 cm year^-1^ diameter growth rate. Solid back line represents the population-wide growth rates based on a hossfeld-IV function, *R*^2^ of the model fit is shown in the figure pane. Note the variation in *y*-axis and *x*-axis scaling.

Growth rates and growth patterns were variable among species, both within and among sites (**Figure 2**). Also within species a high variation in growth patterns of individual trees can be observed, as expressed by the fan shape of the graphs in **Figure 2**. *Brachystegia* spp., *Daniellia, Sweetia*, and *Chukrasia* were the species with the lowest juvenile diameter growth rates (around 3-4 mm year^-1^; **Figure 2**). It took 20–40 years for 75% of the individuals of these species to reach 5 cm dbh (**Table 2**). This is contrasted by the high juvenile growth rates (∼20 mm year^-1^) of the long-lived pioneers *Schizolobium* and *Melia* for which 75% of the individuals had reached 5 cm dbh in 4 years (**Table 2**; **Figure 2**). To account for this time to reach 5 cm dbh, we used the ‘age at 5 cm dbh distribution’ as a threshold value for all further analyses. Applying such a threshold was necessary to exclude a period of apparent regeneration failure as a result of only sampling trees >5 cm dbh. For example, out of the 102 *Cariniana* trees in this study 75% had reached 5 cm dbh in ≤17 years. So, for *Cariniana* all analysis of age distributions was restricted to age classes >17 years.

### Age Distributions

**FIGURE 3:**
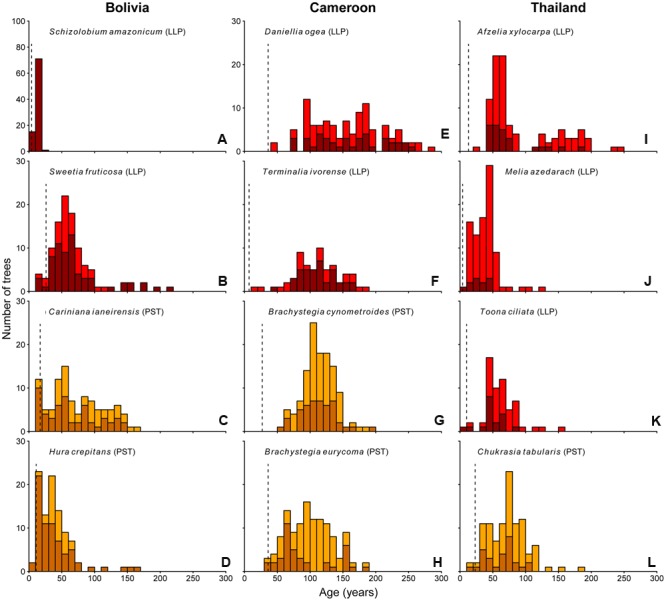
**Age histograms for the 12 study species.** Vertical dashed line indicates the age at which >75% of the individuals in our dataset had reached 5 cm dbh (minimum sampling size used in this study). Shade-tolerance guild is indicated: LLP, long-lived pioneer (red bars); PST, partial shade-tolerant (orange bars). Dark colored bars represent ages of those individuals for which the pith was included in at least one of the tree cores. Lighter colored bars represent those individuals for which the pith was not included in any of the samples.

The age distributions of the 12 study species are shown in **Figure 3**. Of these 12 species, nine show irregular age distributions. In particular, the tree populations in Cameroon and Thailand, with the exception for *Melia*, show a nearly complete absence of young (<30-year-old) trees, with median estimated trees ages of 115 and 58 years, respectively.

**FIGURE 4:**
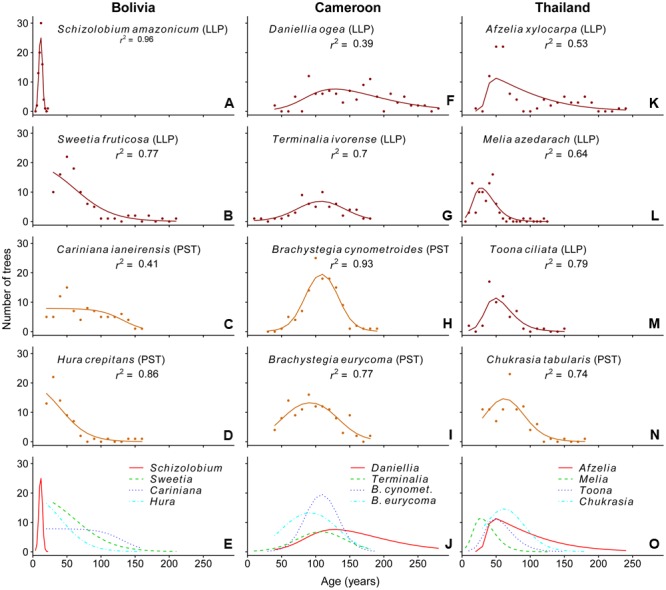
**Observed (dots) and predicted (line) age distributions for the 12 study species.** The line represents the best-fit model based on the AICc-value. The *r*^2^-values shown in the graphs are calculated as the square of the *Pearson’s* correlation coefficient between observed and predicted values. Analysis was restricted to the age classes at which >75% of the individuals in our dataset had reached 5 cm dbh. Except for the bottom panels, the shade-tolerance guild is indicated: LLP, long-lived pioneer (red); PST, partial shade-tolerant (orange).

In Bolivia, where median tree age was 43 years, the age distributions of three species, a long-lived pioneer and two partial shade-tolerants, were best described by exponential and logistic decrease functions, indicating relatively continuous regeneration rates over the past century (**Figures 4B–D,E**). Only the age distribution of the pioneer *Schizolobium* was indicative of an irregular regeneration pattern (**Figure 4A**). All these trees have established in the late 1990s, with mean year of establishment 1998. This establishment peak co-occurs with an exceptionally severe wildfire hitting the region toward the end of the dry-season in 1995 (Pinard et al., 1999; Lopez et al., 2012).

In Cameroon, the population age distributions of the long-lived pioneer *Daniellia* and *Terminalia* were best described by unimodal functions. Both of these species show little recruitment into the younger age classes over periods of 30 and 60 years, respectively, when corrected for the minimum sampling size (**Figures 4F,G**). The age distribution of *Daniellia* was relatively flat (**Figure 4F**), compared to the other three species, because individuals recruited over a longer period (**Table 2**). The age distributions of the two partial shade-tolerant species, *B. cynometroides* and *B. eurycoma*, were similarly unimodal with a large cohort of trees ∼100 years old (**Figures 4H,I**) and a period of absence of regeneration for 50 and 10 years, respectively. Overall the peak in tree ages coincides for the four species at ∼100 years BP (**Figure 4J**).

Age distributions of the four Thai species were also best described by unimodal functions. Although the age distribution of the long-lived pioneer *Afzelia* resembled a bi-modal structure, with two periods of high regeneration around 60 and 160 years ago (**Figure 3I**), this bi-modal shape was not confirmed by the model fitting (**Figure 4K**). We found evidence for reduced recent regeneration rates in all Thai species, but the period of low regeneration was variable per species. The long-lived pioneers *Afzelia* and *Toona*, were characterized by a period of low regeneration of 20 and 30 years (**Figures 4K,M**). *Melia* and *Chukrasia* showed lower rates of recent regeneration, but no marked absence of recent recruits (**Figures 4L,N**). The peak in tree ages coincides, at least for three out of four species, at ∼50 years BP (**Figure 4O**).

## Discussion

To our knowledge this is the first study that investigates regeneration rates of tropical tree species at centennial timescales and over areas of several hundred hectares. We found evidence for recent regeneration failure of long-lived pioneer and partial-shade tolerant tree species at each of the three sites. Particularly in Cameroon and Thailand a large fraction of the species showed virtually no regeneration into the size classes above 5 cm diameter over the past 10–60 years. The irregular age distribution and presence of regeneration pulses of long-lived pioneer and partial shade-intolerant tree species is likely associated with past occurrence of large canopy openings. In the paragraphs below we discuss the most likely factors explaining the observed patterns for each of the sites.

### Disturbance Reconstruction at the Bolivian Site

There are several indications that canopy disturbances have affected the forest stand at the site in Bolivia. The recent establishment pulse of the shade-intolerant *Schizolobium* in Bolivia is indicative of a canopy disturbance in the late 1990s. This was likely a disturbance with a diffuse character, because the trees are distributed across the entire study area and there was no evidence of widespread mortality among canopy trees. The age of the oldest tree (23 years) is unlikely to be close to the maximum age of *Schizolobium*, because the largest tree in our dataset (*n* = 87) had a dbh of 59 cm (Supplementary Figure S2A), whereas in a nearby permanent sample plot several *Schizolobium* trees >100 cm dbh have been found (IBIF, *unpublished results*). The regeneration pulse may have resulted from a large wildfire that occurred toward the end of the dry season in 1995 across an estimated 1 × 10^6^ ha of forest in eastern Bolivia, killing around 40% of the trees, but with a much larger impact on tree saplings than on large canopy trees (Pinard et al., 1999; Lopez et al., 2012). At another seasonal tropical forest site, Baker et al. (2008) showed that low-intensity fires can generate a landscape-scale pulse of canopy gap formation.

In addition to these recent fire-induced disturbances, there is abundant evidence, including charcoal and pottery fragments, of high human population densities in La Chonta in pre-Colombian times (Paz-Rivera and Putz, 2009). Abandonment of former farmlands and settlement may have favored establishment of shade-intolerant tree species. However, human population decline must have occurred >330 years ago (Paz-Rivera and Putz, 2009), which outdates by far the oldest tree that we found, a 220-year-old *Sweetia*. Therefore, age distributions of our study species do not reflect this period of forest recovery. The recent establishment peak of the highly shade-intolerant *Schizolobium* and continuous regeneration rates of the other shade-intolerant species suggest that disturbances, such as fire or drought, were leading to spatially diffuse patterns of gap formation over the past century.

### Evidence for Disturbance in the Cameroonian Site

Age distributions of all four species from the site in Cameroon suggest recent regeneration failure. *Terminalia* is a long-lived pioneer species and an indicator species for secondary forest stands (Lemmens et al., 2012). Successful establishment of such a tree species is dependent on large canopy openings generating adequately high light-levels at the forest floor (Newbery et al., 2013). The three other study species are classified as partial shade-tolerant to shade-intolerant (Hawthorne, 1995). But given the shade-intolerant nature of the juveniles of all four Cameroonian study species, their past regeneration success was likely related to relatively higher light-levels at the forest floor some 100–150 years ago. This situation is contrasted by the presently dense and homogenous canopy of mainly Caesalpinioideae species in the study area, creating strong shading of the forest understory. This dark understory is the most likely cause for the observed low recent regeneration rates of the four study species in the area, because their seedlings all require some canopy opening to survive. Our findings are consistent with observations that non-regenerating long-lived pioneer species are a dominant feature of West African forests (Newbery and Gartlan, 1996; Poorter et al., 1996). These earlier studies hypothesized that an establishment pulse following past disturbance might explain this pattern (see also: Bourland et al., 2015).

Windthrow occasionally flattens areas of up to 1 ha in the nearby Korup National Park, but hurricanes do not occur in the area (Chuyong et al., 2004). Drought and/or human activity are therefore the most likely drivers of canopy disturbance at this site. In their study, Newbery et al. (2004) related establishment of the large canopy tree *Microberlinia bisulcata* (Fabaceae) in the adjacent forest area of Korup National Park to periods of intense drought around 1820–1830 and 1870–1895. The latter period coincides with the period of highest establishment of the four species in this study. These 19th century droughts may have resulted in a period of scattered canopy disturbance (cf. Phillips et al., 2009), favoring establishment of shade-intolerant tree species. In addition, there is also evidence of human occupation of what now seems pristine forests in West and Central Africa (Malhi et al., 2013). This past human occupation was inferred from soil layers containing charcoal and pottery fragments (White and Oates, 1999; Bourland et al., 2015). As in Bolivia, the sudden abandonment of agricultural fields may have induced the establishment success of shade-intolerant tree species at the Cameroonian site. However, we lack any direct evidence on dated charcoal or pottery fragments from our study site to support this hypothesis.

### Evidence for Disturbance in the Thai Site

High past regeneration rates of long-lived pioneer tree species such as *Afzelia, Melia*, and *Toona* are indicative of past large-scale and/or intense canopy disturbance at the Thai site. The 1950s establishment pulse of *Afzelia* was synchronous with establishment of nearly all *Toona* individuals in the area. Intense windstorms that infrequently pass through the region are the most likely causes for heavy canopy disturbance in HKK (Baker et al., 2005) and may have led to the establishment of long-lived pioneer species.

More recent establishment of the shade-intolerant *Melia*, which successfully regenerated well into the late 1980s, is evidence for additional canopy disturbance. These disturbances could be related to low-intensity fires that occurred nearly every year somewhere within HKK over the past two decades (Baker and Bunyavejchewin, 2009). Juveniles of *Afzelia* are, however, noticeably susceptible to understory fires (So et al., 2010) and their successful regeneration may require a situation of both high light and low fire frequency (Vlam et al., 2014a). This combination of rare intensive and abundant extensive disturbances may have caused the irregular tree age distributions observed in the Thai site.

### Site-Level Comparison

In all three sites we found evidence for long-term changes in the regeneration patterns of shade-intolerant species, suggesting the presence of large-scale disturbances that occurred over the past 200 years. In general, the age distributions of trees in the Thai and Cameroonian site indicated more favorable establishment conditions ∼50 and ∼100 years BP, respectively. Based on tree ages alone, however, we cannot conclude whether the unimodal age distributions resulted from historical establishment peaks or recent regeneration failure in combination with accumulated mortality risk (cf. Johnson et al., 1994). Only for *Afzelia* we can confidently conclude that an establishment peak occurred ∼60 years BP, because the typical longevity of the tree is much longer, as can be inferred from the second peak in the age distribution at ∼160 years. The apparent lack of recent regeneration in Cameroon and Thailand is contrasted by the Bolivian site, in which we found higher rates of recent regeneration and a notable recent establishment peak of one pioneer species. Altogether this indicated higher rates of recent disturbance in the Bolivian site than in the other two study sites. Earlier findings of irregular tree-diameter distributions in intact tropical forest stands (Poorter et al., 1996; Bunyavejchewin et al., 2003) and a dominance of long-lived, shade-intolerant tree species in the forest canopy (Newbery et al., 2013), are consistent with such legacies of past severe canopy disturbances.

## Conclusion

Insight into long-term tropical forest stand development will contribute to a better understanding of possible mechanisms generating trends in forest biomass (e.g., Baker et al., 2004) and tropical tree growth (e.g., Groenendijk et al., 2015; van der Sleen et al., 2015). Recovery from past disturbance may induce trends in biomass accumulation and tree growth that cannot be distinguished from those generated by external drivers (Brienen et al., 2016; but see: van der Sleen et al., 2016), like increasing temperatures and increasing atmospheric CO_2_ levels (Chave et al., 2008). Our results show that long-term tropical forest dynamics are driven not only by small-scale disturbances resulting from single treefall gaps, but by a complex history of disturbance regimes varying in scale and intensity.

## Author Contributions

MV as the lead author contributed to the design of the study, acquisition of the data and analysis and interpretation. He wrote the manuscript and integrated suggestions by the other authors to the manuscript. PS and PG were responsible for part of the data collection, study design, interpretation of the data and writing of the manuscript. PZ contributed to the design of the study, analysis of the data and writing the manuscript.

## Conflict of Interest Statement

The authors declare that the research was conducted in the absence of any commercial or financial relationships that could be construed as a potential conflict of interest.

## References

[B1] Agren J. Zackrisson O. (1990). Age and size structure of *Pinus sylvestris* populations on mires in central and northern Sweden. *J. Ecol.* 78 1049–1062. 10.2307/2260951

[B2] Allen C. D. Macalady A. K. Chenchouni H. Bachelet D. Mcdowell N. Vennetier M. (2010). A global overview of drought and heat-induced tree mortality reveals emerging climate change risks for forests. *For. Ecol. Manag.* 259 660–684. 10.1016/j.foreco.2009.09.001

[B3] Baker P. J. Bunyavejchewin S. (2009). “Fire behavior and fire effects across the forest landscape of continental Southeast Asia,” in *Tropical Fire Ecology: Climate Change, Land Use and Ecosystem Dynamics* ed. Cochrane M. A. (Heidelberg: Springer-Praxis) 311–334.

[B4] Baker P. J. Bunyavejchewin S. Oliver C. D. Ashton P. S. (2005). Disturbance history and historical stand dynamics of a seasonal tropical forest in western Thailand. *Ecol. Monogr.* 75 317–343. 10.1890/04-0488

[B5] Baker P. J. Bunyavejchewin S. Robinson A. P. (2008). The impacts of large-scale, low-intensity fires on the forests of continental South-east Asia. *Int. J. Wildland Fire* 17 782–792. 10.1071/WF07147

[B6] Baker T. R. Phillips O. L. Malhi Y. Almeida S. Arroyo L. Di Fiore A. (2004). Increasing biomass in Amazonian forest plots. *Philos. Trans. R. Soc. B Biol. Sci.* 359 353–365. 10.1098/rstb.2003.1422 PMC1693327 15212090

[B7] Bourland N. Cerisier F. Daïnou K. Smith A. L. Hubau W. Beeckman H. (2015). How tightly linked are *Pericopsis elata* (Fabaceae) patches to anthropogenic disturbances in Southeastern Cameroon? *Forests* 6 293–310. 10.3390/f6020293

[B8] Brienen R. J. W. Zuidema P. A. (2003). *Anillos de Crecimiento de Árboles Maderables en Bolivia: su Potencial Para el Manejo de Bosques y una Guia Metodológica. Informe Tecnico N° 7.* Riberalta: Programa manejo de bosques de la Amazonia Boliviana.

[B9] Brienen R. J. W. Gloor M. Ziv G. (2016). Tree demography dominates long-term growth trends inferred from tree rings. *Glob. Chang. Biol.* 10.1111/gcb.13410 [Epub ahead of print]. PMC6849721 27387088

[B10] Brokaw N. V. L. (1985). Gap-phase regeneration in a tropical forest. *Ecology* 66 682–687. 10.2307/1940529

[B11] Bunyavejchewin S. Lafrankie J. V. Baker P. J. Davies S. J. Ashton P. S. (2009). *Forest Trees of Huai Kha Khaeng Wildlife Sanctuary.* Bangkok: National Parks, Wildlife and Plant Conservation Department.

[B12] Bunyavejchewin S. Lafrankie J. V. Baker P. J. Kanzaki M. Ashton P. S. Yamakura T. (2003). Spatial distribution patterns of the dominant canopy dipterocarp species in a seasonal dry evergreen forest in western Thailand. *For. Ecol. Manag.* 175 87–101. 10.1016/S0378-1127(02)00126-3

[B13] Burslem D. F. R. P. Whitmore T. C. Brown G. C. (2000). Short-term effects of cyclone impact and long-term recovery of tropical rain forest on Kolombangara, Solomon Islands. *J. Ecol.* 88 1063–1078. 10.1046/j.1365-2745.2000.00517.x

[B14] Chave J. Condit R. Muller-Landau H. C. Thomas S. C. Ashton P. S. Bunyavejchewin S. (2008). Assessing evidence for a pervasive alteration in tropical tree communities. *PLoS Biol.* 6:e45 10.1371/journal.pbio.0060045 PMC2270308 18318600

[B15] Chuyong G. B. Condit R. Kenfack D. Losos E. C. Nsanyi Moses S. Songwe N. C. (2004). “Korup forest dynamics plot, Cameroon,” in *Tropical Forest Diversity and Dynamism: Findings from a Large-Scale Plot Network* eds Losos E. C. Leigh E. G. (Chicago, IL: University of Chicago Press) 506–516.

[B16] Cirad Forestry Department (2008). *TROPIX 6.0.* Available at: http://tropix.cirad.fr/en [accessed 13-08 2013]

[B17] Clark D. B. Clark D. A. Oberbauer S. F. (2010). Annual wood production in a tropical rain forest in NE Costa Rica linked to climatic variation but not to increasing CO2. *Glob. Change Biol.* 16 747–759. 10.1111/j.1365-2486.2009.02004.x

[B18] Daïnou K. Bizoux J. P. Doucet J. L. Mahy G. Hardy O. J. Heuertz M. (2010). Forest refugia revisited: NSSRs and cpDNA sequences support historical isolation in a wide-spread African tree with high colonization capacity, *Milicia excelsa* (Moraceae). *Mol. Ecol.* 19 4462–4477. 10.1111/j.1365-294X.2010.04831.x 20854478

[B19] Denslow J. S. (1980). Gap partitioning among tropical rainforest trees. *Biotropica* 12 47–55. 10.2307/2388156

[B20] Detienne P. Oyono F. Durrieu De Madron L. Demarques B. Nasi R. (1998). “L’analyse de cernes: applications aux études de croissance de quelques essences en peuplements naturels de forêt dense africaine,” in *Série FORAFRI* (Montpellier: CIRAD-Forêt).

[B21] Elliott S. Kuarak C. Navakitbumrung P. Zangkum S. Anusarnsunthorn V. Blakesley D. (2002). Propagating framework trees to restore seasonally dry tropical forest in northern Thailand. *New For.* 23 63–70. 10.1023/A:1015641119271

[B22] Eschtruth A. K. Battles J. J. (2014). Ephemeral disturbances have long-lasting impacts on forest invasion dynamics. *Ecology* 95 1770–1779. 10.1890/13-1980.1 25163111

[B23] Fraver S. Jonsson B. G. Jönsson M. Esseen P. A. (2008). Demographics and disturbance history of a boreal old-growth *Picea abies* forest. *J. Veg. Sci.* 19 789–798. 10.3170/2008-8-18449

[B24] Gärtner H. Nievergelt D. (2010). The core-microtome: a new tool for surface preparation on cores and time series analysis of varying cell parameters. *Dendrochronologia* 28 85–92. 10.1016/j.dendro.2009.09.002

[B25] Groenendijk P. Sass-Klaassen U. Bongers F. Zuidema P. A. (2014). Potential of tree-ring analysis in a wet tropical forest: a case study on 22 commercial tree species in Central Africa. *For. Ecol. Manag.* 323 65–68. 10.1016/j.foreco.2014.03.037

[B26] Groenendijk P. Van Der Sleen P. Vlam M. Bunyavejchewin S. Bongers F. Zuidema P. A. (2015). No evidence for consistent long-term growth stimulation of 13 tropical tree species: results from tree-ring analysis. *Glob. Change Biol.* 21 3762–3776. 10.1111/gcb.12955 25917997

[B27] Hawthorne W. D. (1995). *Ecological Profiles of Ghanian forest trees.* Oxford: Oxford Foresttry Institute.

[B28] Herwitz S. R. (1993). Growth rates of selected Australian tropical rainforest tree species under controlled conditions. *Oecologia* 96 232–238. 10.1007/BF00317736 28313419

[B29] Huisman J. Olff H. Fresco L. F. M. (1993). A hierarchical set of models for species response analysis. *J. Veg. Sci.* 4 37–46. 10.2307/3235732

[B30] Jansen F. Oksanen J. (2013). How to model species responses along ecological gradients–Huisman–Olff–Fresco models revisited. *J. Veg. Sci.* 24 1108–1117. 10.1111/jvs.12050

[B31] Johnson E. A. Miyanishi K. Kleb H. (1994). The hazards of interpretation of static age structures as shown by stand reconstructions in a Pinus contorta–*Picea engelmannii* forest. *J. Ecol.* 82 923–931. 10.2307/2261455

[B32] Kaewkrom P. Gajaseni J. Jordan C. F. Gajaseni N. (2005). Floristic regeneration in five types of teak plantations in Thailand. *For. Ecol. Manag.* 210 351–361. 10.1016/j.foreco.2005.02.048

[B33] Lebamba J. Vincens A. Maley J. (2012). Pollen, vegetation change and climate at Lake Barombi Mbo (Cameroon) during the last ca. 33 000 cal yr BP: a numerical approach. *Clim. Past* 8 59–78. 10.5194/cp-8-59-2012

[B34] Lemmens R. H. M. J. Louppe D. Oteng-Amoako A. A. (2012). *Plant Resources of Tropical Africa 7 (2) Timbers 2.* Wageningen: PROTA Foundation / CTA.

[B35] Lopez L. Villalba R. Peña-Claros M. (2012). Determining the annual periodicity of growth rings in seven tree species of a tropical moist forest in Santa Cruz, Bolivia. *For. Syst.* 21 508–514. 10.5424/fs/2012213-02966

[B36] Lorimer C. G. (1980). Age structure and disturbance history of a southern Appalachian virgin forest. *Ecology* 61 1169–1184. 10.2307/1936836

[B37] Malhi Y. Adu-Bredu S. Asare R. A. Lewis S. L. Mayaux P. (2013). African rainforests: past, present and future. *Philos. Trans. R. Soc. B Biol. Sci.* 368 10.1098/rstb.2012.0312 PMC3720030 23878339

[B38] Middendorp R. S. Vlam M. Rebel K. T. Baker P. J. Bunyavejchewin S. Zuidema P. A. (2013). Disturbance history of a seasonal tropical forest in western Thailand: a spatial dendroecological analysis. *Biotropica* 45 578–586. 10.1111/btp.12051

[B39] Mostacedo B. Justiniano M. J. Toledo M. Fredericksen T. S. (2003). *Guía Dendrológica de Especies Forestales de Bolivia.* Santa Cruz de la Sierra: BOLFOR.

[B40] Nchanji A. C. Plumptre A. J. (2001). Seasonality in elephant dung decay and implications for censusing and population monitoring in south-western Cameroon. *Afr. J. Ecol.* 39 24–32. 10.1111/j.1365-2028.2001.00265.x

[B41] Nelson B. W. Kapos V. Adams J. B. Oliveira W. J. Braun O. P. G. Do Amaral I. L. (1994). Forest disturbance by large blowdowns in the Brazilian Amazon. *Ecology* 75 853–858. 10.2307/1941742

[B42] Nepstad D. C. Tohver I. M. David R. Moutinho P. Cardinot G. (2007). Mortality of large trees and lianas following experimental drought in an amazon forest. *Ecology* 88 2259–2269. 10.1890/06-1046.1 17918404

[B43] Newbery D. M. Alexander I. J. Rother J. A. (1997). Phosphorus dynamics in a lowland African rain forest: the influence of ectomycorrhizal trees. *Ecol. Monogr.* 67 367–409. 10.2307/2963460

[B44] Newbery D. M. Gartlan J. S. (1996). A structural analysis of rain forest at Korup and Douala-Edea, Cameroon. *Proc. R. Soc. Edinb. B Biol. Sci.* 104 177–224.

[B45] Newbery D. M. Van Der Burgt X. M. Moravie M. A. (2004). Structure and inferred dynamics of a large grove of *Microberlinia bisulcata* trees in central African rain forest: the possible role of periods of multiple disturbance events. *J. Trop. Ecol.* 20 131–143. 10.1017/S0266467403001111

[B46] Newbery D. M. Van Der Burgt X. M. Worbes M. Chuyong G. B. (2013). Transient dominance in a central African rain forest. *Ecol. Monogr.* 83 339–382. 10.1890/12-1699.1

[B47] Nock C. A. Geihofer D. Grabner M. Baker P. J. Bunyavejchewin S. Hietz P. (2009). Wood density and its radial variation in six canopy tree species differing in shade-tolerance in western Thailand. *Ann. Bot.* 104 297–306. 10.1093/aob/mcp118 19454592 PMC2710901

[B48] Nock C. A. Metcalfe D. J. Hietz P. (2016). Examining the influences of site conditions and disturbance on rainforest structure through tree ring analyses in two Araucariaceae species. *For. Ecol. Manag.* 366 65–72. 10.1016/j.foreco.2016.02.008

[B49] Oliver C. D. (1980). Forest development in North America following major disturbances. *For. Ecol. Manag.* 3 153–168. 10.1016/0378-1127(80)90013-4

[B50] Oliver C. D. Larson B. C. (1996). *Forest Stand Dynamics.* New York, NY: Wiley.

[B51] Paz-Rivera C. Putz F. E. (2009). Anthropogenic soils and tree distributions in a lowland forest in Bolivia. *Biotropica* 41 665–675. 10.1111/j.1744-7429.2009.00521.x

[B52] Peña-Claros M. Peters E. M. Justiniano M. J. Bongers F. Blate G. M. Fredericksen T. S. (2008). Regeneration of commercial tree species following silvicultural treatments in a moist tropical forest. *For. Ecol. Manag.* 255 1283–1293. 10.1016/j.foreco.2007.10.033

[B53] Peña-Claros M. Poorter L. Alarcãn A. Blate G. Choque U. Fredericksen T. S. (2012). Soil effects on forest structure and diversity in a moist and a dry tropical forest. *Biotropica* 44 276–283. 10.1111/j.1744-7429.2011.00813.x

[B54] Phillips O. L. Aragão L. E. O. C. Lewis S. L. Fisher J. B. Lloyd J. López-González G. (2009). Drought sensitivity of the amazon rainforest. *Science* 323 1344–1347. 10.1126/science.1164033 19265020

[B55] Pinard M. A. Putz F. E. Licona J. C. (1999). Tree mortality and vine proliferation following a wildfire in a subhumid tropical forest in eastern Bolivia. *For. Ecol. Manag.* 116 247–252. 10.1016/S0378-1127(98)00447-2

[B56] Poorter L. Bongers F. Van Rompaey R. S. A. R. De Klerk M. (1996). Regeneration of canopy tree species at five sites in West African moist forest. *For. Ecol. Manag.* 84 61–69. 10.1016/0378-1127(96)03736-X

[B57] Poorter L. Bongers L. Bongers F. (2006). Architecture of 54 moist-forest tree species: traits, trade-offs, and functional groups. *Ecology* 87 1289–1301. 10.1890/0012-9658200687[1289:AOMTST]2.0.CO;2 16761607

[B58] R Core Team (2013). *R: A Language and Environment for Statistical Computing.* Vienna: R Foundation for Statistical Computing.

[B59] So T. Theilade I. Dell B. (2010). Conservation and utilization of threatened hardwood species through reforestation - An example of Afzelia xylocarpa (Kruz.) *Craib and Dalbergia cochinchinensis Pierre in Cambodia*. *Pac. Conserv. Biol.* 16 101–116. 10.1071/PC100101

[B60] Splechtna B. E. Gratzer G. Black B. A. (2005). Disturbance history of a European old-growth mixed-species forest - A spatial dendro-ecological analysis. *J. Veg. Sci.* 16 511–522. 10.1658/1100-9233200516[511:DHOAEO]2.0.CO;2

[B61] Tanner E. V. J. Rodriguez-Sanchez F. Healey J. R. Holdaway R. J. Bellingham P. J. (2014). Long-term hurricane damage effects on tropical forest tree growth and mortality. *Ecology* 95 2974–2983. 10.1890/13-1801.1

[B62] Toledo M. Poorter L. Peña-Claros M. Alarcón A. Balcázar J. Chuviña J. (2011). Patterns and determinants of floristic variation across lowland forests of Bolivia. *Biotropica* 43 405–413. 10.1111/j.1744-7429.2010.00711.x

[B63] van der Sande M. T. Zuidema P. A. Sterck F. (2015). Explaining biomass growth of tropical canopy trees: the importance of sapwood. *Oecologia* 177 1145–1155. 10.1007/s00442-015-3220-y 25634307 PMC4363484

[B64] van der Sleen P. Groenendijk P. Vlam M. Anten N. P. Bongers F. Zuidema P. A. (2016). Trends in tropical tree growth: re-analyses confirm earlier findings. *Glob. Chang. Biol.* 10.1111/gcb.13572 [Epub ahead of print]. 27865028

[B65] van der Sleen P. Groenendijk P. Vlam M. Anten N. P. Boom A. Bongers F. (2015). No growth stimulation of tropical trees by 150 years of CO2 fertilization but water-use efficiency increased. *Nat. Geosci.* 8 24–28. 10.1038/ngeo2313

[B66] Vandermeer J. De La Cerda I. G. Boucher D. Perfecto I. Ruiz J. (2000). Hurricane disturbance and tropical tree species diversity. *Science* 290 788–791. 10.1126/science.290.5492.788 11052939

[B67] Vlam M. (2014). *Forensic Forest Ecology: Unraveling the Stand History of Tropical Forests.* Ph.D. thesis, Wageningen University Wageningen.

[B68] Vlam M. Baker P. J. Bunyavejchewin S. Mohren G. M. J. Zuidema P. A. (2014a). Understanding recruitment failure in tropical tree species: insights from a tree-ring study. *For. Ecol. Manag.* 312 108–116. 10.1016/j.foreco.2013.10.016

[B69] Vlam M. Baker P. J. Bunyavejchewin S. Zuidema P. A. (2014b). Temperature and rainfall strongly drive temporal growth variation in Asian tropical forest trees. *Oecologia* 174 1449–1461. 10.1007/s00442-013-2846-x 24352845

[B70] Westphal C. Tremer N. Oheimb G. V. Hansen J. Gadow K. V. Härdtle W. (2006). Is the reverse J-shaped diameter distribution universally applicable in European virgin beech forests? *For. Ecol. Manag.* 223 75–83. 10.1016/j.foreco.2005.10.057

[B71] White L. J. T. Oates J. F. (1999). New data on the history of the plateau forest of Okomu, southern Nigeria: an insight into how human disturbance has shaped the African rain forest. *Glob. Ecol. Biogeogr.* 8 355–361. 10.1046/j.1365-2699.1999.00149.x

[B72] Williams L. J. Bunyavejchewin S. Baker P. J. (2008). Deciduousness in a seasonal tropical forest in western Thailand: interannual and intraspecific variation in timing, duration and environmental cues. *Oecologia* 155 571–582. 10.1007/s00442-007-0938-1 18188604

[B73] Willis K. J. Gillson L. Brncic T. M. (2004). How “virgin” is virgin rainforest? *Science* 304 402–403. 10.1126/science.1093991 15087539

[B74] Worbes M. Junk W. J. (1989). Dating tropical trees by means of 14C from bomb tests. *Ecology* 70 503–507. 10.2307/1937554

[B75] Zuidema P. A. Baker P. J. Groenendijk P. Schippers P. Sleen P. Vlam M. (2013). Tropical forests and global change: filling knowledge gaps. *Trends Plant Sci.* 18 1360–1385. 10.1016/j.tplants.2013.05.006 23809291

