# Supplementary material for: Tree Age Distributions Reveal Large-Scale Disturbance-Recovery Cycles in Three Tropical Forests
Source: Front Plant Sci. 2017 Jan 5;7:1984. doi: 10.3389/fpls.2016.01984 (PMC5214098; doi:10.3389/fpls.2016.01984)
Supplement: Supplementary file 1 [file Data_Sheet_1.DOCX]

Supplementary Material

Tree age distributions reveal large-scale disturbance-recovery cycles in three tropical forests

Mart Vlam*, Peter van der Sleen, Peter Groenendijk and Pieter A. Zuidema

*** Correspondence:** Mart Vlam: mart.vlam@wur.nl

**Supplementary Figure 1.** Study area (top) and wood characteristics of the 12 study species (bottom). Approximate location of the study areas is indicated by the star symbol. White arrows indicate tree-ring boundaries. Figure adapted from van der Sleen et al. (2015).

**Supplementary Figure 2.** Diameter distributions for the 12 study species. Shade-tolerance guild is indicated: LLP, long-lived pioneer (red bars); PST, partial shade-tolerant (orange bars).

**References**

Van Der Sleen, P., Groenendijk, P., Vlam, M., Anten, N.P., Boom, A., Bongers, F., Pons, T.L., Terburg, G., and Zuidema, P.A. (2015). No growth stimulation of tropical trees by 150 years of CO2 fertilization but water-use efficiency increased. *Nature geoscience* 8**,** 24-28.
